# Circadian Rhythm and Sleep Disturbances in Young Adult Athletes: A Review About Risk Factors, Consequences, and Interventions

**DOI:** 10.3390/brainsci16020212

**Published:** 2026-02-11

**Authors:** Anne M. Fink, Michele Kerulis

**Affiliations:** 1 Department of Biobehavioral Nursing Science, College of Nursing, University of Illinois Chicago, Chicago, IL 60612, USA; 2 Counseling Department, The Family Institute, Northwestern University, Evanston, IL 60201, USA

**Keywords:** athletes, circadian, mental health, schedules, sleep, sports, stress

## Abstract

**Background/Objectives**: College student athletes can experience sleep and circadian rhythm disturbances. **Methods**: A PRISMA-based systematic review about young adult athletes’ sleep and circadian rhythms was conducted, with 41 published studies analyzed. **Results**: Studies suggest that extending sleep duration could enhance athletic performance and support mental health. Risk factors for sleep and circadian rhythm disturbances include early morning practice sessions, late night games, jet lag, and female sex. Consequences of inadequate sleep include reduced reaction times and mental health problems, such as depressive symptoms and anxiety. Across the studies, numerous research design limitations reduced scientific rigor and hindered the ability to test hypotheses about sleep, circadian rhythms, athletic performance, and mental health outcomes. For example, most studies were underpowered due to small sample sizes and missing data. Many studies lacked randomization, control groups, and objective measures of sleep. Researchers commonly failed to control for variables that could confound results (e.g., caffeine, diet, and menstrual cycle hormones). **Conclusions**: Recommendations for future directions include conducting randomized clinical trials to test interventions related to sleep patterns, nutrition, light exposure, training schedules, and cognitive behavioral therapies to enhance sleep quality. Evidence-based education programs about healthy sleep are essential for coaches and athletes.

## 1. Introduction

College student athletes are vulnerable to disturbances in sleep and circadian rhythms, which may adversely affect their scholastic and athletic achievements and contribute to poor mental health. Commonly, student athletes participate in early morning or night practice sessions, and many competitive sports require travel across time zones [1,2,3]. Chronically disrupted sleep can adversely affect health and performance because cognition, learning, and memory depend on healthy sleep patterns [4]. Inadequate sleep may also hinder recovery after sport-related injuries and contribute to mood disorders such as depression [5,6].

When circadian rhythm disruptions become chronic and severe, athletes could develop a circadian rhythm sleep disorder (CRSD). With this condition, sleep schedules are chronically misaligned with the central nervous system’s intrinsic 24 h rhythms [7]. Examples of CRSDs include Jet Lag Disorder (i.e., caused by traveling across time zones), Irregular Sleep–Wake Rhythm Disorder (i.e., a person’s sleeping and waking times become unpredictable), and Delayed or Advanced Sleep–Wake Phase Disorders (i.e., characterized by difficultly aligning sleeping times to accommodate required practices, competitions, and classes). These disorders cause people to experience excessive fatigue while also suffering from insomnia [7]. Educators, coaches, clinicians, and college administrators require knowledge about healthy sleep and circadian rhythms so they can identify risk factors and prevent adverse outcomes from insufficient sleep. To develop recommendations to improve college student athlete health, we conducted a systematic review of research studies to analyze findings about risk factors, consequences, and potential interventions to prevent circadian rhythm and sleep disturbances, specific to athletes between the ages of 18 and 25 years.

## 2. Materials and Methods

**Figure 1:**
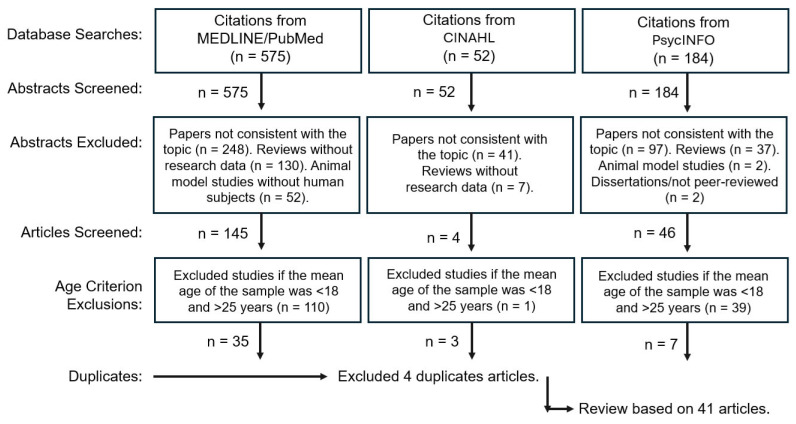
Search strategy for article selection.

The review was conducted according to the 2020 Preferred Reporting Items for Systematic Reviews and Meta-Analyses Guidelines [8]. Literature searches were conducted from August 2025 to January 2026 using the keywords (Circadian Rhythm) AND (Sleep) AND (Sports) in three databases: MEDLINE/PubMed, the Cumulative Index to Nursing and Allied Health Literature (CINAHL), and the American Psychological Association’s (APA) PsycINFO. As illustrated in Figure 1, we excluded review papers, clinical trial protocols (without data), and studies without human subjects. The remaining abstracts were screened to select publications that reported quantitative or qualitative data about sleep- and circadian rhythm-related variables (e.g., using data from actigraphy, polysomnography, sleep logs or questionnaires, core body temperature fluctuations, and melatonin or cortisol levels). Considering that CRSDs are a severe manifestation that is under-diagnosed, we did not require study participants to have any specific diagnoses. Finally, we screened full publications to select studies where the mean or median age of participants was between 18 and 25 years. We selected this age restriction to ensure that the review findings would be relevant to college students (i.e., most subjects were enrolled in college or were elite/professional athletes within the typical college student age range).

Forty-one studies met the criteria for review; study findings and limitations were analyzed by completing Critical Appraisal Skills Program (CASP) checklists that corresponded with each type of research design (e.g., randomized controlled trial [RCT]) [9]. Table 1 summarizes the sample characteristics, key variables, and main study limitations (according to the CASP checklist findings) for each article [10,11,12,13,14,15,16,17,18,19,20,21,22,23,24,25,26,27,28,29,30,31,32,33,34,35,36,37,38,39,40,41,42,43,44,45,46,47,48,49,50]. The present review focuses on each study’s limitations for the purpose of determining future approaches for more rigorous research to inform policies and procedures that support the health of student athletes.

## 3. Results

Collectively, the studies provided important insights into young adult athletes around the world, focusing on their experiences with sleep and circadian rhythm disturbances, as well as their mental health and athletic performance. As shown in Table 1, the articles reported findings from RCTs, observational cross-sectional (‘OBS Cross’), or longitudinal/cohort studies (‘OBS Cohort’). When researchers examined various types of sleep-manipulating interventions, the interventions could be classified into four categories: (1) protocols causing prolonged or reduced sleep [10,30,33,35,41,42], (2) light exposure [22,23,36,50], (3) food and supplement interventions [14,31,43], and (4) altitude-induced changes (hypoxia exposure during sleep) [40]. In addition, seven studies examined possible sex and gender differences in sleep patterns, circadian rhythms, and athletic performance [17,25,27,28,29,35,44].

### 3.1. Effects of Prolonged or Reduced Sleep Duration on Athletic Performance

By manipulating athletes’ sleep, researchers investigated associations among athletic performance, sleep timing, and sleep duration. Data from sleep logs and actigraphy recordings demonstrated how many athletes habitually obtained 6 to 8 h of sleep a night [11,12,13,16,23,27,34,37,38,46,49]. Only one study measured athletic performance after extending sleep [33], and another study determined whether daytime napping altered sleep during the following night [11]. Other investigations were designed to examine outcomes after sleep restriction [30,42] or sleep deprivation [35,41]. When college athletes slept significantly longer (increasing sleep duration by 1.4 ± 0.7 h [*p* < 0.001] to extend the total duration from 8 h to 10 h]), they demonstrated greater standing broad jump distances (*p* < 0.001) and faster reaction times on the Psychomotor Vigilance Test (*p* = 0.03) [33]. Napping was also examined as an intervention for increasing sleep—Boukhris et al. compared subjects randomized to daytime nap opportunities (comparing 25 versus 90 min naps in a cross-over design). Neither nap duration impaired nighttime sleep architecture [11]. The total sleep duration, rapid eye movement (REM) sleep, and non-REM sleep parameters were statistically similar across the control (no nap), 25 min nap, and 90 min nap conditions. It was not possible to determine whether napping had a beneficial effect on athletic performance, however, because Boukhris et al. did not include any athletic assessments [11].

Reducing sleep led to quantifiable impairments in athletic performance in several studies [21,30,35,41,42]. For example, in rugby players, Skein et al. found that although muscle strength did not differ significantly between the normal sleep and sleep deprivation conditions, other performance metrics were significantly impaired by the lack of sleep; for example, a reduction in countermovement jump distances and slower reaction times were observed. In addition, Skein et al. found significantly higher levels of serum biomarkers that indicated muscle damage (creatinine kinase) and inflammation (C-reactive protein) after sleep deprivation [41]. Souissi et al. measured the effects of reduced sleep on the next day’s strength and anaerobic capacity before and after 5 min of judo combat [42]. First, when the athletes who practiced judo (i.e., judokas) slept undisturbed in a laboratory setting (sleeping from 11 p.m. to 6 a.m.), performance data from the following day’s judo combat revealed a diurnal variation in muscle strength—judokas demonstrated significantly more strength in the afternoon compared with the morning. Then, judokas were randomized to experience sleep deprivation during the beginning of the night (sleeping from 3 a.m. to 6 a.m.) or during the end of the night (sleeping from 11 p.m. to 3 a.m.); both sleep deprivation conditions eliminated the diurnal pattern in strength during judo combat the next day. Sleep deprivation at the end of the night was associated with significant decrements in strength the following afternoon [42]. Souissi et al. concluded that early rising may be more detrimental than late bedtimes, particularly when competitions are scheduled for the afternoon. This study did not include any objective measures of subjects’ chronotype or sleep architecture, however, which limits our ability to understand how sleep variables may have correlated with judo combat performance [42].

In a randomized crossover experiment, Nishida et al. compared golf performance after restricting sleep to 4–5 h compared with normal/habitual sleep (7 h). In this study, reduced sleep was associated with poorer accuracy, which was defined as the distance between the gold ball and the target (golf cup) measured in centimeters [30]. The golfers’ scores on the Morningness–Eveningness Questionnaire also predicted accuracy during afternoon training for the subjects who were classified as ‘morning types’ (preferred earlier waking times), which underscored how chronotype preferences may interact with sleep duration to affect athletes’ performance [30]. In a laboratory setting, Kline et al. observed swimmers for 50 consecutive hours as they adhered to repeated ‘ultra-short’ 3 h sleep–wake cycles (1 h of sleep in darkness and 2 h of wakefulness in dim light) [21]. Each swimmer performed six 200 m swim trials eight times a day (each separated by 9 h). To analyze circadian variations in swim performance, swim times were z-transformed and compared over time against the cosine of intra-aural temperature data. The swimmers demonstrated optimal/peak swimming performance five to seven hours before their lowest body temperature; their poorest swim performance occurred within an hour of the lowest intra-aural temperature. The difference between the optimal and poorest performance was approximately 5.8 s, which is important during an athletic competition [21].

Not all studies reported results linking reduced sleep with poorer athletic performance or injuries [10,12]. For example, Burke et al. monitored sleep using wrist actigraphy during the football season, finding no significant relationship between sleep duration and athletes’ risk for injury [12]. In another study about football players, Abedelmalek et al. randomized a small sample (*n* = 36) to compare Wingate test scores after normal sleep versus after an early wake-up time (sleeping from 10:30 p.m. to 3 p.m.); the Wingate Fatigue Index did not differ significantly according to the sleep conditions [10].

### 3.2. Chronotypes, Mental Health, and Sport Schedules

Chronotypes refer to people’s natural tendencies and preferences for sleeping at earlier versus later times. Data from six of the reviewed studies indicated that assessing young adults’ chronotypes may be important for developing interventions that promote athletic achievements while optimizing mental health [18,20,27,28,46,47]. When rugby players had early morning training sessions (7 a.m.), they slept 6.7 ± 1.4 h (compared with 8.9 ± 2.1 h when they did not have events scheduled in the morning), leading the study authors to conclude that early training schedules may cause inconsistent sleep patterns [48]. When comparing time of day and performance in a 5-week retrospective analysis of pre-season basketball performance, Heishman et al. found enhanced performance during the afternoon (e.g., longer countermovement jump distances [59 ± 1 cm in the morning versus 62 ± 2 cm in the afternoon]), concluding that when coaches need to optimize sleep in their players, avoiding early morning training sessions may prolong sleep and enhance athletic abilities [16]. Sargent et al. also reported significant associations among early morning training sessions, reduced sleep duration, and high pre-training self-reported fatigue [37]. Similarly, Facer-Childs and Brandstaetter emphasized the importance of coaches’ assessments of young adult athletes’ chronotypes to understand peak performance and rest times, although they did not measure sleep [15]. Using a randomized cross-over design, Vitale et al. tested the hypothesis that soccer players’ sleep quality would differ after high-intensity interval training (occurring at 8:00 a.m. or 8:00 p.m.) depending on whether a player reported a morning or an evening chronotype, but with a small sample size (*n* = 23), they did not find statistically significant differences [46].

In observational studies where actigraphy monitors were utilized to measure athletes’ sleep patterns, sleep duration was significantly reduced after games that occurred in the late evening [19,38]. For example, Juliff et al. demonstrated that night games were followed by shorter sleep duration and lower sleep efficiency in netball players [19]. Importantly, this study also included neuroendocrine assays (salivary cortisol and urinary adrenaline levels) and psychological trait questionnaires (Hyperarousal Scale). Playing a night game was associated with shorter sleep and early awakening; salivary cortisol levels and core temperatures were not significantly different compared with a rest day, but urinary noradrenaline levels rose after a night game (234 ± 137 nmol/L [rest day: 89 ± 54 nmol/L]). Juliff et al. did not find urinary noradrenaline levels to correlate with any sleep parameters. Considering that scores on the Hyperarousal Scale correlated with sleep efficiency (r = −0.60, *p* < 0.01), Juliff et al. concluded that athletes’ perceived psychological stress may be a predictor of reduced sleep, even when physiological biomarkers like urinary catecholamines do not fluctuate significantly [19].

Two studies about athletes’ chronotypes revealed significant associations between sleep/wake schedules and mental health variables [18,47]. Hu et al. used validated questionnaires to estimate the prevalence of mental health problems among competitors during the Beijing 2022 Winter Olympics (7-item Generalized Anxiety Disorder Questionnaire, 9-item Patient Health Questionnaire, and 7-item Insomnia Severity Index), which revealed depressive symptoms in 20%, anxiety in 15%, and insomnia in 13% of respondents [18]. An evening chronotype was a significant predictor of reporting symptoms of depression, anxiety, and insomnia. Although this study lacked measures of athletic performance, Hu et al. concluded that misaligned sleep schedules and chronotype preferences may predispose athletes to develop mental health problems, and coaches should consider these factors during preparatory and recovery periods [18]. Wills et al. studied the relationship between evening chronotypes and depressive symptoms in university basketball players, hypothesizing that staying awake late may contribute to social isolation [47]. Using questionnaires (Circadian Energy Scale, Center for Epidemiological Studies Depression Scale, and the Multidimensional Scale of Perceived Social Support), Wills et al. found that players with evening chronotypes reported significantly higher depressive symptoms, but only if they also perceived inadequate support from family, friends, and teammates. These findings indicated how mental health interventions for young adult athletes must be tailored to improve sleep health and social support [47]. According to Tan et al., athletes’ behaviors, such as caffeine intake and the use of electronic devices before bedtime, are also important considerations for improving sleep and mental health [44]. Data from Wilson et al. emphasized the importance of tailoring information about sleep to consider and respect cultural and religious traditions—Wilson et al. monitored sleep diaries in soccer players before, during, and after Ramadan [49]. They found that when athletes were fasting and praying in observance of Ramadan, betimes were significantly later, total sleep duration was longer, and urine osmolality increased (825 ± 143 mOsmol/kg versus 708 ± 155 mOsmol/kg). Even though sleep and hydration were altered during Ramadan, training loads did not differ over time [49].

### 3.3. Travel and Schedule Considerations—Circadian Phase Shifts

Five investigations examined the effects of jet lag caused by travel across time zones; four of these studies collected data during athletic competitions [24,26,40,45], and one study was conducted in a controlled laboratory setting [32]. During the World Rowing Junior Championships, Kölling et al. measured sleeping habits and subjective jet lag (Liverpool John Moore’s University Jet-Lag Questionnaire) when rowers had an 11 h westward flight that caused a 5 h time shift [24]. Rowers responded by sleeping earlier and significantly longer for the first two nights after the flight; however, they continued to report jetlag symptoms for six days. This study did not include any objective measures of athletic performance—aside from competition outcomes (e.g., boat placings)—therefore, it was not possible to analyze relationships between jet lag symptoms and athletic skills [24]. Using data from the International Cricket Council Women’s World Cup, Lalor et al. determined whether sleeping during international flights could reduce jet lag symptoms [26]. Eleven cricketers traveled westward for approximately 19 h, and their in-flight sleep duration was determined by actigraphy (14.6 ± 3.6 h [mean ± standard deviation]). Cricketers with a higher in-flight sleep efficiency (% of time asleep) reported lower subjective ratings of fatigue (Likert scale) and less muscle soreness during the tournament period [26].

Athletes demonstrated an ability to adapt their sleep to new time zones to preserve athletic performance [32,45], although some athletes remained susceptible to sleepiness and fatigue [40]. When Varesco et al. studied speed skaters who traveled from Canada to Asia for the World Cup competition, they found that skaters naturally prolonged their sleep by 9 min per night (over 5 days) before returning to their habitual sleep duration. Considering that the sample’s sleep duration and countermovement jump height increased during the competition phase (*n* = 10), Varesco et al. interpreted this finding as an indication that elite athletes can adapt and manage jet lag when they have additional time to sleep [45]. When female gymnasts completed questionnaires before World Cup competitions, scores indicating poor quality sleep (Pittsburgh Sleep Quality Index) predicted daytime sleepiness ratings (Epworth Sleepiness Scale) [40]. Gymnasts who reported high levels of daytime sleepiness had significantly greater stress levels (Sport Competition Anxiety Test-A) and poorer scores during competitions [40]. To simulate the effects of jet lag, Petit et al. studied a 5 h phase shift in athletes in a laboratory setting [32]. The subjects underwent polysomnography and core temperature recordings to evaluate sleep and circadian rhythms. In this sample, shifting the circadian phase 5 h ahead caused a reduction in total sleep time with less REM sleep and less stage 2 non-REM sleep. The 5 h phase shift was associated with higher blood lactate levels after exercise; however, athletes performed the Wingate test without significant reductions in exercise capacity [32].

To test the hypothesis that muscle strength demonstrates diurnal and circadian patterns, Robertson et al. recruited 10 male weight-lifters [34]. Their sample size was supported by a power analysis to demonstrate that 10 subjects were sufficient to detect differences between day versus night strength metrics (80% power; 5% two-sided significance error level) [34]. They compared results (bench press and leg exercises) for four times, namely 3:00 a.m., 9:00 a.m., 3:00 p.m., and 9:00 p.m., with subjects resting for at least 72 h between sessions. Cosinar analyses demonstrated circadian variations in weight-lifting abilities; for example, peak torque and peak velocity were highest in the evening. In this study, however, subjects were not randomly assigned to exercise times. The results suggest that coaches and exercise physiologists should consider how ‘time of day’ could affect muscle physiology (e.g., excitation contraction coupling), as this information could inform future recommendations for training and competition times for different types of sports [34].

### 3.4. Food, Melatonin, and Light Therapy Interventions

Several investigations were conducted to determine whether nutritional interventions, melatonin supplementation, or light exposure could alter athletic performance or mental health. When nutritional interventions were examined, the researchers did not standardize the dosages of any nutrients or supplements, which hindered the ability to rigorously research the effects. For example, Takeuchi et al. compared groups of soccer players who were given different instructions for eating breakfast—one group was told to eat foods rich in tryptophan and vitamin B6 (e.g., soybeans and bananas) and then expose themselves to sunlight after breakfast (light exposure duration unknown) for four weeks (group sample size was not reported). They compared this group’s Morningness–Eveningness Questionnaire scores with two other groups: (1) soccer players who consumed tryptophan and vitamin B6 for breakfast but did not increase sunlight exposure and (2) soccer players who were not given instructions to change their diets or increase sunlight exposure [43]. Takeuchi et al. reported findings suggesting that the combined dietary and sunlight intervention could ‘shift’ participants from an evening to a morning chronotype; however, the study had too many limitations to test this hypothesis (e.g., group assignments were not randomized, meals and light exposure were not standardized, and the group sample sizes were not reported) [43]. In another study, Doherty et al. examined Pittsburgh Sleep Quality Index (PSQI) scores during a 6-week study to determine whether consuming kiwi fruit (contains melatonin) an hour before bedtime altered self-reported assessments of sleep [14]. Although Doherty et al. concluded that kiwifruit consumption correlated significantly with improved PSQI sleep quality scores and fewer awakenings from sleep, the study did not include randomization, objective sleep architecture measures, melatonin assays, or a control group [14].

Only one study included a consistent and standardized dosage of melatonin [31]. Paryab et al. investigated the effects of melatonin supplements on athletes after normal sleep, sleep restriction (sleep duration reduced by 4 h), and 24 h of sleep deprivation [31]. Subjects received a placebo or melatonin (6 mg) 30 min before undergoing testing to determine static and dynamic balance, reaction times, and anaerobic power. Sleep restriction and deprivation had significant adverse effects on these athletic performance measures. When subjects received 6 mg of melatonin, the sleep restriction group demonstrated improvements in balance, anaerobic power, reaction time, and blood lactic acid levels (*p* < 0.05). Melatonin supplementation did not improve any metrics in the group that underwent 24 h of sleep deprivation [31].

Considering hypotheses about dim/red light to promote melatonin secretion from the pineal gland, several research teams tested light therapies as potential methods for manipulating sleep and athletic performance. For example, Zhao et al. tested the effects of red light on sleep quality and endurance in basketball players. They compared whole-body exposure to red light therapy (30 min nightly for 14 days) to outcomes from a control group (placed in the same supine position in the light chamber without any light illumination). The red light therapy was associated with significant increases in serum melatonin levels (from baseline), improved subjective ratings of sleep quality (Pittsburgh Sleep Quality Index), and greater 12 min run distances (pre- and post-intervention differences within the experimental group) [50]. During the Rio 2016 Olympic Games, Rosa et al. tested an 8-day bright/green-blue light intervention to entrain swimmers’ sleep/wake cycles and potentially improve their abilities for nighttime competition (∼30 min of green-blue light exposure between 6 p.m. to 8 p.m. each evening) [36]. During the 8-day protocol, the swimmers slept significantly longer on the third, seventh, and eighth days (*p* = 0.01), and Psychomotor Vigilance Tests demonstrated improved reaction times when comparing the first and eighth days. Although these findings demonstrate potential beneficial effects of bright light therapy for improving sleep and athletic performance, because this study lacked randomization, a control group, and the quantification of circadian biomarkers, the data provided limited information about this potential strategy [36].

Another study provided similar findings but was hindered by difficulties with subjects’ protocol adherence—Knufinke et al. tested a light exposure intervention to determine whether a combination of light and sleep schedule training could improve athletes’ sleep and performance [23]. To increase morning light exposure, subjects wore light-emitting goggles, and during the evening their light exposure was reduced by wearing amber-lens glasses. The study utilized a randomized crossover design to examine all subjects under both conditions. Due to low adherence to the protocol, however, Knufinke et al. analyzed the data according to two additional groups—athletes who kept a strict sleep–wake schedule (*n* = 8) and a group with lenient sleep–wake schedules (*n* = 25). In the latter group (lenient schedule), the light regulation intervention reduced the sleep onset latency by approximately 8 min (self-report). In the strict schedule group, sleep onset latency was reduced by 17 min, and the intervention was also associated with improved subjective sleep quality. Actigraphy data, however, did not show any changes associated with the intervention [23]. Another study about light therapy did not have statistically significant results because the light intervention did not correlate with any objectively measured changes in athletic performance [22]. In this study, Knaier et al. conducted a randomized clinical trial to determine whether cyclists’ strength and reaction times could be altered with different evening (9 p.m.) light exposure conditions: (1) bright light (~4400 lux), monochromatic blue light (~230 lux), and a control condition (~230 lux) [22]. Compared with controls, handgrip strength increased by 0.9 kg in subjects exposed to bright light. Handgrip strength decreased by 0.3 kg in subjects exposed to blue light, although neither change was statistically significant. Regarding reaction times, the bright light and blue light differences were also not statistically different (−1 ms and 2 ms, respectively, relative to controls). Data from the groups’ salivary melatonin assays demonstrated that evening light exposure reduced subjects’ pineal gland melatonin secretion (0.4 pg/mL versus 0.9 pg/mL [control]) [22,51].

### 3.5. Autonomic Nervous System Modulations of Heart Rate

Researchers examined autonomic heart rate modulation in two observational cohort studies [13,39]. In a 42-day study of soccer players, Costa et al. collected overnight actigraphy and electrocardiogram data to compare three time periods: sleep after night (9 p.m.) training sessions, sleep after competitions (matches), and sleep on rest days [13]. When subjects slept after their training sessions, the mean (± standard deviation) heart rate was significantly higher (57 ± 1 beats per minute [bpm], *p* < 0.001) compared with rest days (57 ± 1 bpm) or match days (56 ± 1 bpm)—the corresponding mean durations of sleep for each condition were 7 h (±28 min; after night-training), 8 h (±3 min; after matches), and 9 h (±53 min; rest days). Costa et al. also calculated heart rate variability (HRV) metrics, which were log-transformed for comparisons across the three conditions (night-training versus matches versus rest). This analysis detected statistical differences across the time periods for the Root Mean Square of Successive Differences between Normal Heartbeats (RMSSD) and for spectral power in the high-frequency (HF) and low-frequency (LF) bands. For example, two HRV indices that suggest enhanced vagal (parasympathetic) activity (log transformed RMSSD and HF power) increased during the rest periods as subjects experienced reduced stress [13]. Costa et al. interpreted these findings as a recommendation for coaches to consider how training and match schedules affect autonomic cardiac regulation during sleep to ensure that young athletes experience periods of more restorative sleep. Unfortunately, the study did not include objective measures of the athletes sleep architecture; therefore, it was not possible for Costa et al. to determine how slow-wave and REM sleep may have changed under each condition [13].

In a 21-day study about skiing performance, Schmitt et al. tested the hypothesis that sleeping at a high altitude could enhance skiing performance [39]. To examine this hypothesis, skiers were randomized to sleep under hypoxic (FiO_2_ 15%, *n* = 18) or normoxic conditions (FiO_2_ 21%, *n* = 6) for 15 days while the investigators acquired data from treadmill and skiing performance tests after the sleep sessions. Some of the post-test measures indicated improved performance when athletes slept under the hypoxic condition, such as improved VO_2_ max after the first day of hypoxic exposure [39]. In this study, Schmitt et al. also included a group of subjects (*n* = 15) to test daily adjustments in training according to HRV analysis—for example, if a subject demonstrated HRV changes that suggested increased stress (e.g., reduced HF power), they were assigned to lower-intensity training or to a rest day. Despite the limitations posed by the small sample size (*N* = 24) and short study duration (21 days), the findings obtained by Schmitt et al. suggest that attention to sleeping environments and HRV-guided adjustments in training could be utilized by coaches to enhance oxygen consumption and performance in endurance athletes [39].

### 3.6. Sex and Gender Differences

While some of the studies included only male or female athletes (Table 1), which did not allow statistical comparisons according to sex or gender, seven studies compared findings from male and female athletes [17,25,27,28,29,35,44], but only one study included an analysis of the menstrual cycle [17]. When Koutouvakis et al. compared actigraphy recording between male and female water polo players, they found significantly higher nocturnal sleep efficiency in females compared with males (i.e., females reported fewer awakenings from sleep during the night), but the small sample size (*n* = 27) and short study duration (1 week) limited the ability to determine whether sleep differences correlated significantly with athletic outcomes [25]. A cross-sectional analysis of sleep and circadian rhythm questionnaires by Tan et al. revealed that female athletes were more likely to report evening chronotypes (i.e., energy peaks later in the day), while male athletes were more likely to have a morning chronotype (i.e., energy peaks in the morning); however, the study did not include any objective measures of circadian rhythms (e.g., dim light melatonin onset [44]).

In a 2-month study, Hrozanova et al. examined sex differences regarding sleep architecture (contactless radar sleep tracking) and subjective sleep quality (PSQI scores) in winter endurance athletes [17]. They found that female athletes reported poorer sleep quality on the PSQI despite sleeping for a longer period than males. The analysis of sleep architecture demonstrated more N1 sleep and more REM sleep in females, as well as a shorter REM sleep latency. A comparison of the menstrual and pre-menstrual phases indicated that N1 and deep slow-wave sleep were reduced during the menstrual period. Compared with the luteal phase, female athletes demonstrated lower sleep efficiency and increased deep (N3) sleep during the follicular phase of the menstrual cycle. Although this study had a small sample of females (*n* = 15), the findings underscore the need for more research about improving sleep architecture to optimize athletic performance and recovery in larger samples of young female athletes [17].

In another study, Romdhani et al. reported diurnal patterns in agility, which were associated with sleep loss and athletes’ sex. In their study of basketball and tennis players, the female group (*n* = 11) demonstrated greater agility than males (*n* = 11) after 24 h of sleep deprivation. This study did not include any objective quantification of hormonal levels or sleep architecture, however, so it is not possible to understand how the subjects’ sex and sleep affected their athletic responses [35]. When Mascaro et al. studied Australian Rules football players for 2 weeks, they found that female footballers had significantly higher scores on the Athlete Psychological Strain Questionnaire than male players (17.7 ± 7.1 versus 14.1 ± 4.0, *p* < 0.01), indicating high perceived levels of stress. In addition, female players reported later sleep times, and the mean circadian phase (objectively determined by dim light melatonin onset) was significantly later in females than in males (8:47 p.m. versus 8:14 p.m., *p* < 0.05). Based on these findings, Mascaro argued that coaches should be particularly aware of the importance of adequate sleep for female athletes to support their athletic and scholastic performance as well as their mental health [29]. Litwic-Kaminska and Kotysko did not find statistically significant differences in actigraphy metrics or PSQI scores between males and females in their studies because they did not sample sufficient numbers of male and female participants to test research questions about sex or gender [27,28].

## 4. Discussion

The main finding from the systematic review demonstrated that young adult athletes are at risk of sleep and circadian rhythm disturbances due to early morning practice sessions, late night games, and jet lag. Coaches, college administrators, and clinicians who treat young adult athletes should be aware of the potentially serious consequences of disturbed sleep and circadian rhythms—important consequences include mental health problems (e.g., stress, depressive symptoms, and anxiety) and poorer athletic performance. To advance science, it is important to recognize that all of the reviewed studies had critical limitations, which should be analyzed to enhance the rigor of future research. For example, researchers relied on self-reported and subjective measures of sleep that were not confirmed by actigraphy, photoplethysmography, or polysomnography. The present review also demonstrated the need for more rigorous research into potential interventions. Although a few trials were conducted to test sleep-enhancing therapies (e.g., melatonin supplementation or light therapy), the efficacy of these interventions remains unknown because the studies had small sample sizes (low statistical power) and usually lacked rigorous designs (e.g., no control groups). Also, the limited information about sex- and gender-related variables emphasizes the importance of conducting more research about women in sports, especially longitudinal studies to understand relationships among sleep, sex hormone fluctuations, mental health variables, and physical activity and performance.

The literature about sleep and athletes could be advanced by expanding the focus to encompass sleep neuroscience and endocrine biomarkers. Only two studies involved the calculation of HRV metrics; designing future investigations to understand sympathetic and parasympathetic modulation of the heart during sleep and wakefulness could be important for guiding coaches to plan training that optimizes performance while reducing the risk of injuries. For example, Jin et al. proposed using HRV data to monitor and adjust training loads in basketball players [52], which could also include adjustments based on circulating biomarkers, such as blood lactate [53]. In rowing athletes, Sherman et al. found that females demonstrated changes in the RMSSD metric of HRV throughout the menstrual cycle [54], supporting the notion that researching neurobiological and hormonal fluctuations—within the context of athletic performance and sleep—could elucidate new strategies for supporting the health of athletes. In addition, because genetic and behavioral characteristics affect chronotype preferences and health in young adults [55], research into the loci associated with circadian regulation could lead to discoveries that advance knowledge about mental health and performance, metabolism, and future disease risk [56]. Improved knowledge about the behavioral factors that affect sleep in young adults (e.g., caffeine, studying, and electronic device use [44]) could inform future cognitive-behavioral interventions to improve sleep in athletes.

Addressing the limitations of the present systematic review is also important. To focus on a college student athlete population, we chose to exclude articles about younger or older people (<18 or >25 years of age), which reduced the number of studies to analyze. Also, because we excluded articles without any measures of sleep and circadian rhythms, we may have missed data about important correlates of sleep disturbances, such as studies about mental and physical health and future disease risk. The reviewed studies focused on elite athletes, so our findings may not be relevant to the general population with lower fitness levels.

## 5. Conclusions

In conclusion, circadian rhythm biology and sleep science are important components of programs that support the mental and physical health of student athletes. Considering that the scientific literature on this topic has numerous methodological limitations (e.g., small sample sizes, lack of randomization or control/placebo groups, and the omission of objective measures), the first step is to enhance the rigor of sleep research in athletes. Designing future rigorous and reliable studies will inform policies and improvements for athletes’ health to include public education, nutritional interventions, and new technologies.

## Figures and Tables

**Table 1:** Summary of studies—research participants, designs, and limitations.

**Study Citation**	***N***	**Age, Years** **(M ± SD)**	**% Female**	**Sport (Location)**	**Key Variables and Metrics**	**CASP Checklist Summary**	
						**Study Design**	**Study Limitations**
Abedelmalek et al. [10]	36	21 ± 1	N.R.	Football (Tunisia)	Actigraphy, Wingate test	RCT	No power analysis was conducted for sample size determination. Randomization and blinding methods were not reported. Although understanding socioeconomic status (SES) was a study aim, no objective SES measures were included.
Boukhris et al. [11].	14	23 ± 2	50%	N.R. (Australia)	Actigraphy	RCT	The power analysis was based on data from older adults and not on student athletes.
Burke et al. [12].	84	20 ± 2	0%	Football (USA)	Actigraphy, time-loss injury	OBS Cohort	Damaged actigraphy and protocol noncompliance reduced the sample size from 94 to 84 men; data may not be generalizable to female athletes.
Costa et al. [13].	17	21 ± 2	100%	Soccer (Portugal)	Actigraphy, HRV, perceived exertion, training impulse	OBS Cohort	The study did not include important variables, such as menstrual cycle data, food intake, or light exposure, which could affect outcomes.
Doherty et al. [14].	15	23 ± 4	40%	Running or sailing (Ireland)	Recovery Stress Questionnaire	OBS Cohort	Subjects were not randomized to conditions. No control group. Fuit consumption (i.e., melatonin dose) was not standardized.
Facer-Childs & Brandstaetter [15].	20	20	58%	Field hockey (UK)	Aerobic capacity (VO2 max)	OBS Cross	Did not measure sleep objectively. Small sample size.
Heishman et al. [16].	10	21 ± 1	0%	Basketball (USA)	Accelerometer, central nervous system readiness, countermovement jump	OBS Cohort	Results about timing may be biased because basketball drills were only performed in the afternoon, but strength and conditioning sessions occurred periodically according to facility availability (no randomization). Only males were studied. Paired t-tests were calculated without considering potential changes during the 5-week pre-season period.
Hrozanova et al. [17].	56	18 ± 1	34%	Endurance sports (Norway)	Contactless radar sleep tracking, menstrual cycle calendar	OBS Cohort	Subject attrition was an issue (e.g., 7% of subjects dropped out, and menstrual cycle data were obtained from only 15 subjects).
Hu et al. [18].	428	21 ± 4	44%	Winter sports (China)	Questionnaires (e.g., Insomnia Severity Index)	OBS Cross	Data were self-reported without any objective measures of sleep or athletic performance.
Juliff et al. [19].	12	19	N.R.	Netball (Australia)	Actigraphy, core temperature, salivary cortisol, urine catecholamines	OBS Cohort	No power analysis was conducted for sample size determination. No randomization to rest versus night game conditions.
Kentiba et al. [20].	174	22 ± 2	28%	Various (Ethiopia)	Questionnaire (Horne Ostberg Morningness–Eveningness)	OBS Cross	Data were self-reported without any objective measures of sleep or athletic performance.
Kline et al. [21].	25	21 ± 1	52%	Swimming (USAs)	200 m swim, actigraphy, temperature	OBS Cross	The limited data points (i.e., six data points over 50 h) were not sufficient for calculating circadian rhythmicity. Intra-aural temperature may be less reliable than core body temperatures. No power analyses were reported for sample size determination.
Knaier et al. [22].	72	23	0%	Cycling (Switzerland)	Acoustic reaction time, handgrip strength, salivary melatonin	RCT	Although the authors report conducting a power analysis, they did not state the method and required sample. The degree of light intensity was varied.
Knufinke et al. [23].	26	25 ± 3	53%	Various (The Netherlands)	Actigraphy	RCT	Subjects demonstrated low adherence to the sleep schedule protocol; 5 subjects did not complete the protocol, and the study was under-powered.
Kölling et al. [24].	30	18 ± 1	0%	Rowing (Australia)	Actigraphy	OBS Cohort	The study had missing data and lacked a control group for comparison. The subjects had different mealtimes and training schedules.
Koutouvakis et al. [25].	27	22 ± 4	63%	Water Polo	Perceived exertion, training duration	OBS Cohort	The small sample size hindered the ability to determine relationships among athletic performance, sex, and sleep parameters. No power analysis was reported.
Lalor et al. [26].	11	24 ± 3	100%	Cricket (Australia)	Actigraphy, perceived exertion	OBS Cohort	No baseline sleep data were collected before traveling.
Litwic-Kaminska & Jankowski [27].	82	21 ± 2	27%	Various (Poland)	Actigraphy	OBS Cross	No power analyses were reported for sample size determination, especially for testing the aim about sex differences.
Litwic-Kaminska & Kotysko [28].	207	21 ± 2	27%	Various (Poland)	Actigraphy	OBS Cross	No power analyses were reported for sample size determination.
Mascaro et al. [29].	87	24 ± 2	43%	Football (Australia)	Actigraphy, dim-light melatonin onset	OBS Cross	The study had missing data (only 53 subjects provided data for timepoints).
Nishida et al. [30].	11	21 ± 1	100%	Golf (Japan)	Actigraphy, putting accuracy	RCT	The sample size was small and restricted to men. Putting accuracy was determined at home (self-reported) without video confirmation of results.
Paryab et al. [31].	10	20 ± 2	100%	Various (Iran)	Anaerobic power, Stroop test, blood lactic acid levels	RCT	No objective sleep measures were included. The sample size was small, and only one dosage (6 mg melatonin) was tested.
Petit et al. [32].	16	22 ± 2	0%	Various (France)	Aerobic capacity (VO2 max), Wingate test, polysomnography, core temperature, blood lactate levels	RCT	The study provided data for a short period of time (i.e., one lab habituation night, one baseline recording, two interventions a week apart).
Ritland et al. [33].	50	20 ± 2	50%	Military tactical sports (USA)	Actigraphy, executive function, psychomotor vigilance, standing broad jump distance	RCT	No power analyses were reported for sample size determination.
Robertson et al. [34].	10	22 ± 1	0%	Weight-lifting (UK)	Isokinetic and isometric strength, muscle and rectal temperature	OBS Cohort	Subjects were not randomized to start times. Sleep was not objectively measured.
Romdhani et al. [35].	22	21 ± 1	50%	Basketball or Tennis (Tunisia)	Agility, oral temperature, perceived exertion	RCT	Sleep duration was not standardized or measured objectively.
Rosa et al. [36].	22	25 ± 3	50%	Swimming (Brazil)	Actigraphy, reaction time	OBS Cohort	No control group was available for comparisons.
Sargent et al. [37].	70	20 ± 3	35%	Various (Australia)	Actigraphy	OBS Cohort	Observational study could not account for confounders (e.g., caffeine and alcohol use).
Sargent et al. [38].	22	22 ± 3	0%	Football (Australia)	Actigraphy	OBS Cohort	Pre-season data may not be generalizable to competitions.
Schmitt et al. [39].	24	23 ± 4	21%	Skiing (France)	Aerobic capacity (VO2 max), 10 km roller-ski test, HRV, hypoxic duration, oxygen saturation, hemoglobin, ferritin	RCT	No power analysis was conducted for sample size determination. Randomization and blinding methods were not reported.
Silva & Paiva [40].	67	19 ± 3	100%	Gymnasts (Portugal)	Basal metabolic rate	OBS Cross	Without longitudinal data, it was not possible to examine relationships between performance and daytime sleepiness over time. Sleep and alertness were not measured objectively.
Skein et al. [41].	11	20 ± 3	0%	Rugby (Australia)	Countermovement jump, Stroop test, C-reactive protein, creatine kinase	RCT	Small sample size. No objective sleep data were collected.
Souissi et al. [42].	12	19 ± 2	0%	Judo (Tunisia)	Handgrip test, maximal voluntary contraction, perceived exertion, Wingate test	RCT	Small sample size. No objective sleep data were collected. Randomization and blinding methods were not reported.
Takeuchi [43].	83	18 to 22	0%	Soccer (Japan)	Food and lifestyle questionnaires	RCT	Groups were not randomly assigned. Group sample sizes were not reported. Diet and sunlight exposure were not standardized. Statistical analysis details were limited; only non-parametric tests were calculated. The authors mentioned missing data but did not provide information (e.g., degrees of freedom).
Tan et al. [44].	933	19 ± 4	50%	Various (China)	Questionnaires	OBS Cross	No objective sleep measures (all data were self-reported).
Varesco et al. [45].	19	24 ± 4	58%	Speed skaters (Canada)	Actigraphy, countermovement jump	OBS Cohort	No power analyses were reported for sample size determination.
Vitale et al. [46].	23	22 ± 2	29%	Soccer (Italy)	Actigraphy	RCT	The authors reported that “sleep quality was poorer” in morning-type subjects after evening training, but no data from actigraphy was provided to support this conclusion. Sleep parameter data are not reported in the paper. A larger sample was recruited (*N* = 547), but only 23 subjects completed the RCT. The number of female subjects was only reported for the total sample but not for the 23 subjects who completed the RCT.
Wills et al. [47].	189	19 ± 5	46%	Basketball (USA)	Questionnaires	OBS Cross	Study only included self-report measures.
Wilson et al. [48].	21	21 ± 2	0%	Rugby (United Kingdom)	Actigraphy, questionnaires	OBS Cohort	No power analysis was conducted for sample size determination.
Wilson et al. [49].	20	25 ± 2	0%	Soccer (Qatar)	Aerobic capacity (VO2 max), core temperature (disposable temperature sensor pills), urine osmolality	OBS Cohort	Small sample size; only two subjects provided core body temperature data. No mental health variables were included.
Zhao et al. [50].	20	19 ± 4	100%	Basketball (China)	Cooper 12 min run, serum melatonin levels	RCT	No power analysis was conducted for sample size determination. No objective sleep measures were included.

## Data Availability

No new data were created or analyzed in this study. Data sharing is not applicable to this article.

## Abbreviations

The following abbreviations are used in this manuscript:

CRSD	Circadian rhythm sleep disorder
HF	High frequency
HRV	Heart rate variability
N.R.	Not reported
OBS	Observational study
PSQI	Pittsburgh Sleep Quality Index
REM	Rapid eye movement
RMSSD	Root Mean Square of Successive Differences between Normal Heartbeats
RTC	Randomized controlled trial
